# Portable wireless neurofeedback system of EEG alpha rhythm enhances memory

**DOI:** 10.1186/s12938-017-0418-8

**Published:** 2017-11-13

**Authors:** Ting-Ying Wei, Da-Wei Chang, You-De Liu, Chen-Wei Liu, Chung-Ping Young, Sheng-Fu Liang, Fu-Zen Shaw

**Affiliations:** 1 Department of Computer Science and Information Engineering, National Cheng Kung University, Tainan, Taiwan; 2 Institute of Medical Informatics, National Cheng Kung University, Tainan, Taiwan; 3 Department of Psychology, National Cheng Kung University, Tainan, Taiwan; 4 Mind Research and Imaging Center, National Cheng Kung University, Tainan, Taiwan

**Keywords:** Neurofeedback, Alpha rhythm, Memory, Wireless, Bluetooth

## Abstract

**Background:**

Effect of neurofeedback training (NFT) on enhancement of cognitive function or amelioration of clinical symptoms is inconclusive. The trainability of brain rhythm using a neurofeedback system is uncertainty because various experimental designs are used in previous studies. The current study aimed to develop a portable wireless NFT system for alpha rhythm and to validate effect of the NFT system on memory with a sham-controlled group.

**Methods:**

The proposed system contained an EEG signal analysis device and a smartphone with wireless Bluetooth low-energy technology. Instantaneous 1-s EEG power and contiguous 5-min EEG power throughout the training were developed as feedback information. The training performance and its progression were kept to boost usability of our device. Participants were blinded and randomly assigned into either the control group receiving random 4-Hz power or Alpha group receiving 8–12-Hz power. Working memory and episodic memory were assessed by the backward digital span task and word-pair task, respectively.

**Results:**

The portable neurofeedback system had advantages of a tiny size and long-term recording and demonstrated trainability of alpha rhythm in terms of significant increase of power and duration of 8–12 Hz. Moreover, accuracies of the backward digital span task and word-pair task showed significant enhancement in the Alpha group after training compared to the control group.

**Conclusions:**

Our tiny portable device demonstrated success trainability of alpha rhythm and enhanced two kinds of memories. The present study suggest that the portable neurofeedback system provides an alternative intervention for memory enhancement.

## Background

Biofeedback or neurofeedback is an operant conditioning paradigm to learn how to control physiological behaviors through a series of trial-and-error processes . Neurofeedback provides valuable information derived from real-time brain activity, such as electroencephalography (EEG), and displays the result on an interface of visual, audio, or other modalities. Thus, users can learn to control their brain activities through a neurofeedback apparatus. The control ability of brain rhythms from users often elicits a neural plasticity in the brain then affects their behaviors and cognitive functions as well [1–3].

The alpha rhythm of 8–12 Hz displays in the occipital cortex and its neighboring area under an eye-closed condition. Originally, functional hypothesis of alpha rhythm is related to cortical inhibition [4], which may be crucial to lower brain activity. Neurofeedback training (NFT) of alpha rhythm is employed quite frequently as a treatment for several clinical disorders such as anxiety [5] or depression [6]. However, its effectiveness is still debated in clinic (for review see [7, 8]). In addition to clinical application, NFT of alpha rhythm is also assessed on cognitive function in healthy subjects [2]. Numerous studies show positive effect of the alpha NFT on attention or memory [9, 10]. However some studies have no enhancement in memory or cognitive function with altered amplitude or peak frequency of alpha rhythm [11–13]. Several reasons are crucial for these controversial effects. The trainability for an NFT system is the first issue. Previous studies have indicated a short training session being insufficient for cognitive enhancement [12–14]. Can we develop and validate a possible NFT apparatus for a long-term training with a great usability? That will be very important to increase training sessions of the NFT under laboratory/clinical settings or daily environment.

Additionally, most previous studies are lack of comparable control group [9–11] or absence of the control group [15, 16]. Different experimental designs often cause controversial results in the findings. Identical exposure for a training apparatus and the same protocol between the control and experimental groups are crucial to identify effect of an intervention. To reduce possible selection bias of participants and related internal validity, a sham-controlled experimental design is more appropriate to validate effect of an NFT on memory or cognitive function [12, 17]. Thus, the experimental design with a sham control group is beneficial to determine the NFT effect in a new developed system [18].

Most available neurofeedback systems are laboratory-designed and contain wires to the training machine, resulting in inconvenience or constraints for subjects. Wireless technologies are widely utilized in medical devices and biomedical research [19, 20]. A wireless recording not only improves the system convenience but also reduces the possible artifacts from recording wires [21]. Additionally, the main functions of a neurofeedback training program are easily implemented with a smartphone application. Users can install the application and perform the training procedures on their own devices. Due to the portability brought by the utilization of the smartphone, the proposed system can be easily used in a daily life [22, 23].

The present study aimed to develop a portable neurofeedback training system for subjects to perform trainings in a flexible training environment. The proposed system comprised an EEG signal analysis device that was wirelessly connected to smartphones by Bluetooth low energy wireless technology. The system illustrated interactive information of current alpha power to training subjects. Subjects learned how to produce and control the alpha rhythm as much as possible through visual feedback indexes. Moreover, both working memory and episodic memory before and after the training were evaluated compared with a sham-controlled group to assess performance of the wireless NFT system.

## Methods

Thirty healthy participants (age 26 ± 3 years) from the National Cheng Kung University were blinded and randomly assigned into two groups (i.e., Alpha, n = 15; control, n = 15). All participants were right-handed and had no experience of taking NFT in the past. The two groups had no difference in the factors of gender (p = 0.75), age (p = 0.46), and education (p = 0.39). The entire experiment complied with guidelines and regulations in the Institutional Review Board of National Cheng Kung University Hospital. Informed consent was provided and signed for all participants.

### Hardware architecture

**Fig. 1:**
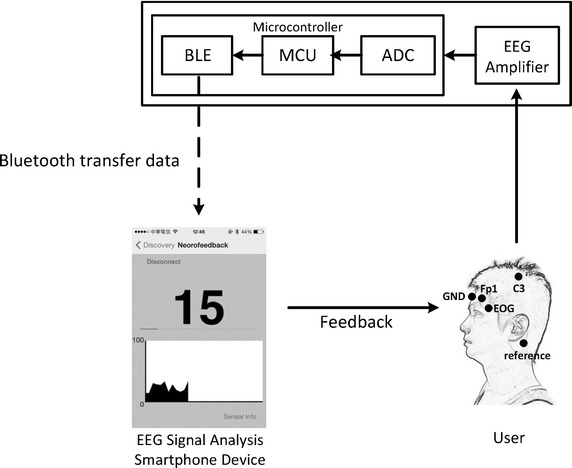
Schematic drawing of the neurofeedback training (NFT) apparatus for user from signal amplification, data transfer, and display on a smartphone through Bluetooth transmission. *ADC* analog-to-digital converter, *BLE* Bluetooth low energy, *EEG* electroencephalogram, *MCU* microcontroller unit

The hardware architecture of our portable neurofeedback system (Fig. 1) primarily contained an EEG amplification board, a microcontroller module, and a smartphone. The EEG amplification board aimed to amplify brain activity. The microcontroller module was responsible for EEG data sampling and to control wireless transmission of a Bluetooth module. The smartphone was used to receive and calculate wireless EEG data as a visual feedback and to save data.

The present study used a single-channel EEG recording through Ag/AgCl electrodes. Based on previous neurofeedback studies [1, 17], we selected a C3 channel as an active lead with a reference over the contralateral mastoid area (M2) according to the 10–20 system [24]. A ground electrode was placed over the Fpz region. The EEG signal was amplified with a gain of 10,000 through an instrumentation amplifier (AD623, Analog Device, Texas) in combination with two non-inverting operational amplifiers (AD8538, Analog Device, Texas) within a frequency range of 0.15–50 Hz [25]. The amplified EEG was then positive-biased to an analog-to-digital converter (ADC) of the microcontroller.

The microcontroller module included a MSP430F5438 integrated chip, which embedded with a MSP430 microcontroller unit, 256 kB flash memory, 16 kB RAM, and other peripherals such as an 8-bit ADC and three 16-bit timers. The MSP430 digitized data through an embedded ADC with 128 Hz and transferred sampled EEG data to a Bluetooth module. Afterwards, the Bluetooth module transmitted the data to a smartphone. The core component of this Bluetooth module was a Nordic nRF8001 chip that integrated a fully compliant Bluetooth radio and link layer controller. Bluetooth is designed for short-range and low power wireless communication, and it is widely adopted in personal computers and consumer electronic devices, e.g., mobile phone or media player. The present study used the Bluetooth version 4.0, which aimed at applications in the fitness, healthcare and security areas because it provided lower cost, lower power consumption, and a comparable communication range than a traditional Bluetooth protocol [20, 23].

### Software implementation

The software of the proposed training system contained two parts: control firmware on the microcontroller module and a training application on the smartphone. The EEG signal analysis device was able to pair with any Bluetooth-compatible mobile device with the training application installed. To reduce effort of porting the proposed system to other mobile devices, all the analysis and calculation in the proposed system was executed on the microcontroller module of the EEG signal analysis device. The firmware running in the microcontroller module performed EEG signal acquisition, data analysis, and wireless transmission. The application running on the smartphone provided a graphic user interface to configure the training procedure and displayed the real-time EEG feedback. The software components were described below.

### Data analysis and wireless transmission of the NFT

**Fig. 2:**
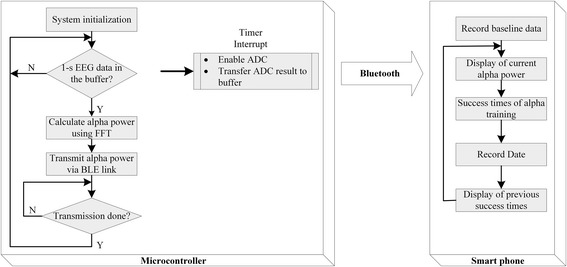
Schematic flowchart of the EEG signals acquisition, data analysis, and wireless transmission between the microcontroller and smartphone through Bluetooth transmission for NFT. *FFT* fast Fourier transform

The data analysis task fetched the 1-s sampled EEG data in the buffer and then performed fast Fourier transform (FFT) to calculate the power of the alpha rhythm. Both raw data and calculated data were transmitted immediately to the smartphone via Bluetooth communication. Figure 2 shows the flowchart of the firmware (left) of the microcontroller, including the main program for EEG acquisition, EEG analysis, and wireless transmission. The smartphone received and displayed the alpha power and the total success duration for 1-s alpha events. Participants saw all training performance in terms of changes of alpha power and alpha duration throughout the training sessions over a smartphone. In addition, information of EEG changes with regard to training number per day displayed on the smartphone at the end of each training session.

**Fig. 3:**
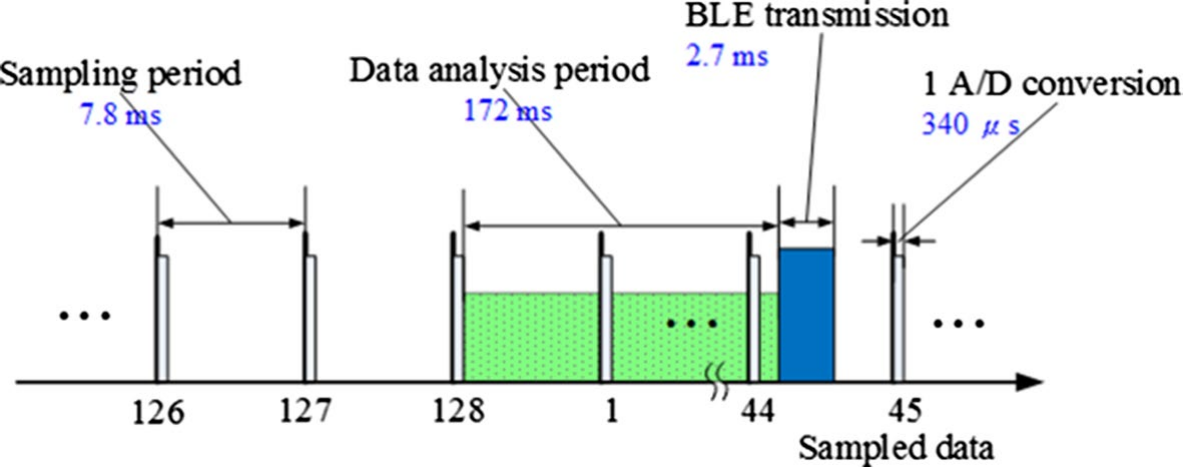
Timing diagram of the EEG signal acquisition, data analysis, and wireless transmission tasks

Figure 3 reveals timing diagram of the EEG signal acquisition, data analysis, and wireless transmission tasks running on the microcontroller unit (MCU). The timing was obtained by toggling an MCU I/O pin at the start and end of the task and measuring the duration via an ADC (USB-6009, National Instruments, TX). The data analysis task was performed every 128 sampling periods. The Bluetooth transmission was triggered immediately after the completion of the data analysis task. The execution time of the data analysis task and the latency for wireless transmission were 172 and 2.7 ms, respectively. Although the data analysis task spanned several sampling periods, sampled data collection always performed with a higher priority.

### Training interface of the NFT

**Fig. 4:**
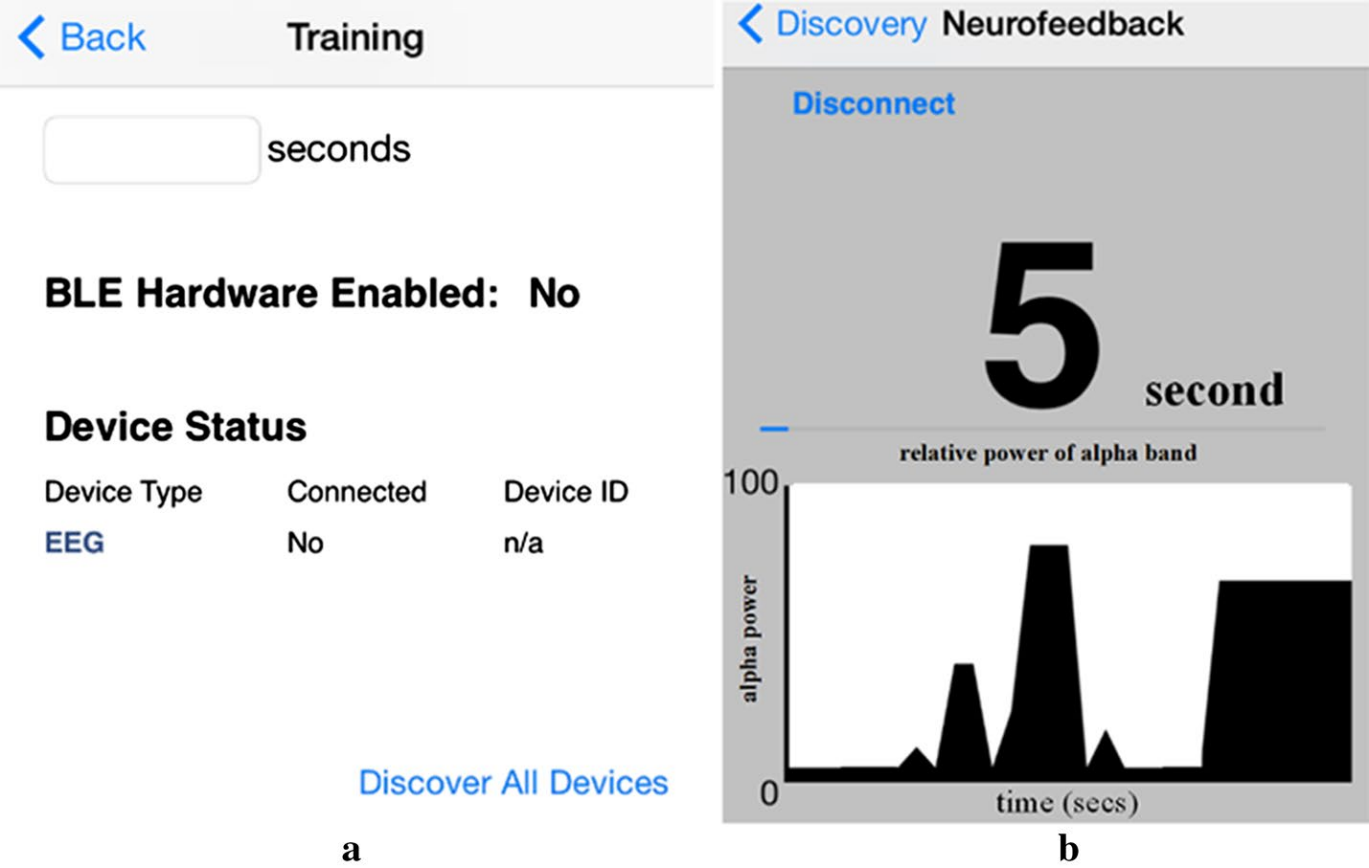
The setup window (**a**) and the visual feedback (**b**) of the neurofeedback training system. Information of the visual feedback contains duration of success alpha episode (top) and alpha power (bottom)

The training application running on the smartphone was utilized for displaying real-time feedback. When the application started, users entered the desired time length of the training procedure and set up a Bluetooth connection between the signal analysis device and the smartphone (Fig. 4a). The connection was set up by clicking the “Discover All Devices” button to search nearby Bluetooth devices. The identification and type of Bluetooth devices displayed in a list to allow a user to select a target for Bluetooth connection setup. Thus, the training procedure was ready. Thereafter, quality of EEG recording was ascertained when the amplitude was < 100 μV_rms_ after properly adjusting electrode–electrolyte-scalp conjunction.

During the training procedure, the training application received real-time consecutive 1-s EEG data wirelessly and displayed the information of calculated alpha power on the screen of the smartphone. Figure 4b shows the information of success episode number, alpha power of the current episode, and changes of consecutive alpha powers. The blue bar of Fig. 4b reflects current alpha power. The waveform shown in the bottom panel represented consecutive alpha powers. The received EEG data was stored in the internal storage of the smartphone for future analysis. Users were able to terminate the training procedure before the end of the procedure by clicking the Disconnect button. At the end of each session, alpha powers and success number of 300-s training episodes displayed on the smartphone to allow user/researcher to develop or establish their strategy through trial-and-error learning [26].

### System assessment

The present study used a 3.7 V, 1000 mAH, Li-ion battery (HYB, China) for the EEG signal analysis device. Current consumption of the EEG signal analysis device was measured using a 6-1/2-digit Digital Multimeter (USB-4065, National Instruments). Operation duration of a Li-ion battery was defined under a free running test until the system ran out of power. The timestamp testing data in the smartphone indicated battery life of the EEG signal analysis device.

### Experimental procedure

To verify the effect of the proposed system on memory, three-stage experiment (i.e., pretest, training, and posttest) was designed. The pretest and posttest of three cognitive tests were performed immediately before and after the training stage. During the training stage, the 1-channel EEG signal (C3-M2) was utilized. Subjects in the Alpha group received the projection of alpha power (8–12 Hz) on the screen of a smartphone. The control group received various randomly selected 4-Hz bandwidth in the range of 7–20 Hz for every 1-s event, which was used in our previous study [26].

At the beginning, brain activity was recorded and analyzed to assess its noise level, including artifacts of eye blink or muscle contraction, etc. To reduce possible artifact signals, each subject was reminded before the training [26]: (1) avoiding frequent eyes blink; (2) eyes closure or fall asleep was informed as an invalid strategy; (3) avoiding body’s movement or shaking/nodding head; (4) avoiding too much facial expression intentionally. A digital camera was used to rule out the effects of these behavioral artifacts.

**Fig. 5:**
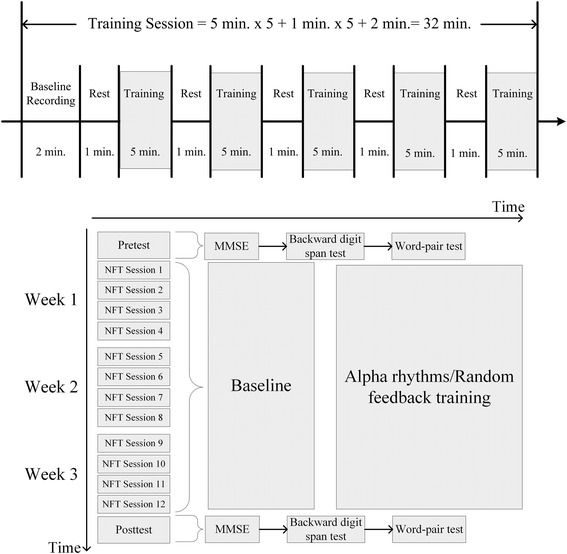
Schematic plot for a training session (top panel) and procedure of the three-phase experiment (bottom panel) in an NFT. The pretest and posttest phases contain three cognitive tasks. Twelve sessions are performed in an NFT. *MMSE* Mini-Mental State Examination

Twelve training sessions were performed within 3 weeks (Fig. 5). Four sessions were performed per week. A session contained 5 blocks, and each block took 5 min. In the beginning of a training, a 2-min baseline EEG was recorded followed by 1-min rest. Thereafter, a 5-min training block followed by a 1-min resting period was performed. Subjects used the proposed system and attempted to increase activities of particular rhythms shown on the screen of a smartphone.

In an NFT, participant can see the instantaneous information of the 1-s power of a selected bandwidth and the waveform of all consecutive 1-s powers of a selected bandwidth. The instantaneous power was expressed in a horizontal bar (Fig. 4b). If an EEG power increased, the bar moves to right side. Otherwise, an EEG power decreased, the bar moved to left side. Participants were instructed to move the bar to the rightmost position and to maintain the bar as long as possible.

During the 1-min rest period between two blocks, we tried to help participants to develop a good strategy using the information of consecutive 1-s power information (the bottom panel of Fig. 4b). For example, we pointed out timestamps with higher power in the training block and asked participants to recall the strategy they used. During the inter-block rest, we encouraged participants to try their best to move/control the bar. Although the control group seemed to be uneasy with controlling their brain activities, they reported no difficulty and frustration during the training.

### Evaluation of cognitive function

The cognitive function was evaluated through the backward digit span test, word pair test, and Mini-Mental State Examination (MMSE). MMSE was used to evaluate possible cognitive impairment. MMSE was able to evaluate various cognitive abilities, such as orientation to time and space, recall, language, attention, calculation, etc. The MMSE score ranged from 0 to 30 points. A MMSE score greater than or equal to 25 points indicated normal cognition [27]. Participant was excluded if MMSE < 25 in this study.

The backward digit span task [28] is a measure of working memory and contains phases of practice and test. In the practice phase, subjects were instructed to familiarize themselves with the processes of the test. In the test phase, thirty trials were performed. At the beginning of each trial, the subject was asked to focus on a cross symbol on the monitor. A series of digits (4–8 randomly) were displayed after the cross disappeared, and each digit lasted for one second. The subject answered the digits in a reverse order on an answer sheet at the end of each trial. Each digit in the correct place had one point. There were a total of 180 digits in the 30 trials, thus the maximum score was 180 points.

The word-pair test [26, 29] was composed of two phases, learning and retrieval phases. In the learning phase, the monitor displayed a cross for 3500 ms followed by a pair of Chinese words for 1500 ms. Thereafter, a white screen was displayed for 5000 ms before the next start. Eighty word pairs were used in the word-pair test. Subjects had a 30-min break between the learning and retrieval phases. In the beginning of the retrieval phase, a cross was displayed for 3500 ms to make the subject focus on the monitor, followed by a priming word for 6500 ms. Subjects had to pronounce the paired word within 6500 ms. Each correct answer was worth 1 point. The maximum score was 80.

### Data analysis

In an NFT, EEG was transferred into a power spectrum using FFT with a Hamming window. Power of the alpha bandwidth or particular bandwidth was obtained by summation of selected bandwidth in the power spectrum. Thereafter, the power was projected to a horizontal bar to indicate current status of EEG (Fig. 4b). To further illustrate time–frequency characteristics of various activities, such as cortical activity of the C3 or Fp1 lead, electrooculogram (EOG), or electromyogram (EMG), a short-time FFT with a Hamming window was performed with 50% data overlapping.

There were two indexes used to assess the training progression of EEG throughout 12 training sessions: the mean alpha power ratio and total duration of successful alpha events [26]. Alpha power ratio is defined by the power of 8–12 Hz normalized by averaged 8- to 12-Hz power of all 1-s baseline EEGs as shown below.

$${\text{Alpha power ratio}} = \frac{Alpha\,power}{Baseline\,alpha\,power}$$

If alpha power ratio of 1-s EEG was higher than 1.2, thus the 1-s EEG segment was considered as a successful event. All successful 1-s events within a session were cumulated as an index of the total duration of successful alpha events. Moreover, alpha power ratios of all successful 1-s EEGs within a session was averaged to obtain an index of the mean alpha power ratio. The mean alpha power ratio throughout 12 sessions was used to reflect dynamic changes of alpha powers within a NFT [26].

### Statistical analysis

Demographic data (age, education, gender) in the two groups were analyzed by independent t test or Chi square test, respectively. The normality and equal variance of the data were assessed for a parametric statistic. Mean alpha power ratio and total alpha duration throughout 12 training sessions in the two groups were analyzed by two-way analysis of variance (ANOVA) with one-factor repetition, if appropriate, followed by t test with Bonferroni correction. Accuracies of the backward digital span task and word-pair task were assessed by two-way ANOVA with one-factor repetition. The temporal relationship in activities of different channels was calculated by Pearson correlation coefficient r. Furthermore, independent t test was used to compare r values between two channels. All statistical analyses were performed by SigmaPlot. Data were expressed as the mean ± standard error of the mean. A two-tailed significance level was set at p < 0.05.

## Results

### System evaluation

Dimensions of the Bluetooth module (6.01 g), EEG amplification board (2.29 g), and MCU board (15.91 g) were 32 × 23 × 6 mm^3^, 48 × 24 × 4 mm^3^, and 45 × 45 × 6 mm^3^, respectively. The Li-ion battery weighed 17.98 g. Total weight of the EEG acquisition device was 60.18 g. Participants had no complaint on the load of the device within the NFT. During an NFT, mean current consumption was 26.24 mA. The battery supported continuous 21-h operation of the device. Because each training procedure typically required > 32 min, the signal analysis device supported > 40 NFT sessions. In general, user can recharge the battery after each NFT.

### Assessment of interference on the device

**Fig. 6:**
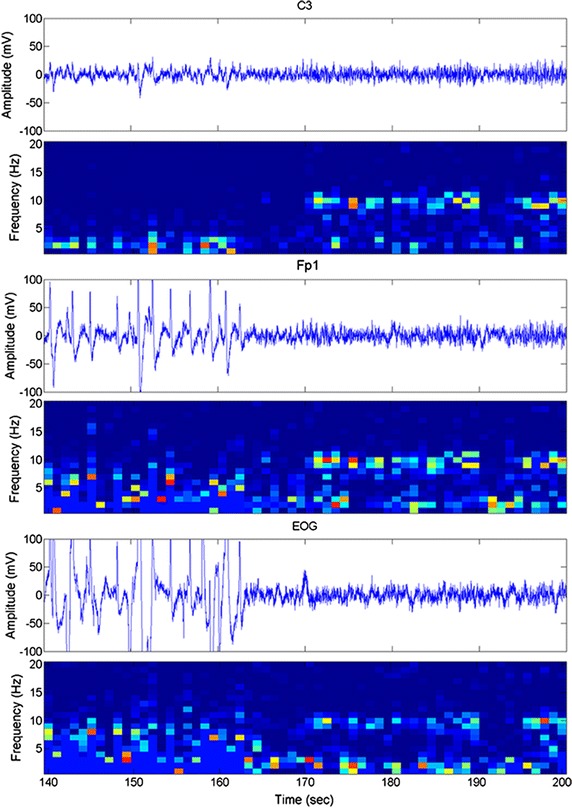
Example of artifact of eye blink in electrooculogram (EOG) and EEGs of the Fp1 and C3 leads accompanied by their time–frequency spectrograms. The trace contains numerous eye blinks at the beginning followed by obvious alpha activation

There were two possible interferences due to eye blink or muscular activity on the NFT. To further ascertain interference on the alpha effect of a C3 lead, we performed recordings of EOG and two EEG leads (Fp1 and C3) simultaneously. Figure 6 illustrates their temporal traces and their time–frequency spectrograms in the Alpha group. The traces of EOG and Fp1 recordings showed numerous and serious eye-blink artifacts at the beginning followed by alpha activation. The artifact of eye blink had a high energy in the low frequency range from 0 to 12 Hz in the EOG and Fp1 traces. In contrast, the C3 lead illustrated low-amplitude eye-blink activity at the beginning of the trace, which was dominant at the frequency range of 0–6 Hz with no obvious influence on the alpha bandwidth. The r value between EOG and the Fp1 lead (0.779 ± 0.238) was significantly higher than that of the C3 lead (0.134 ± 0.36; t = 4.843, *p* < 0.001). The results suggest that the C3 lead has a low interference from eye blink for a training of the alpha band.

**Fig. 7:**
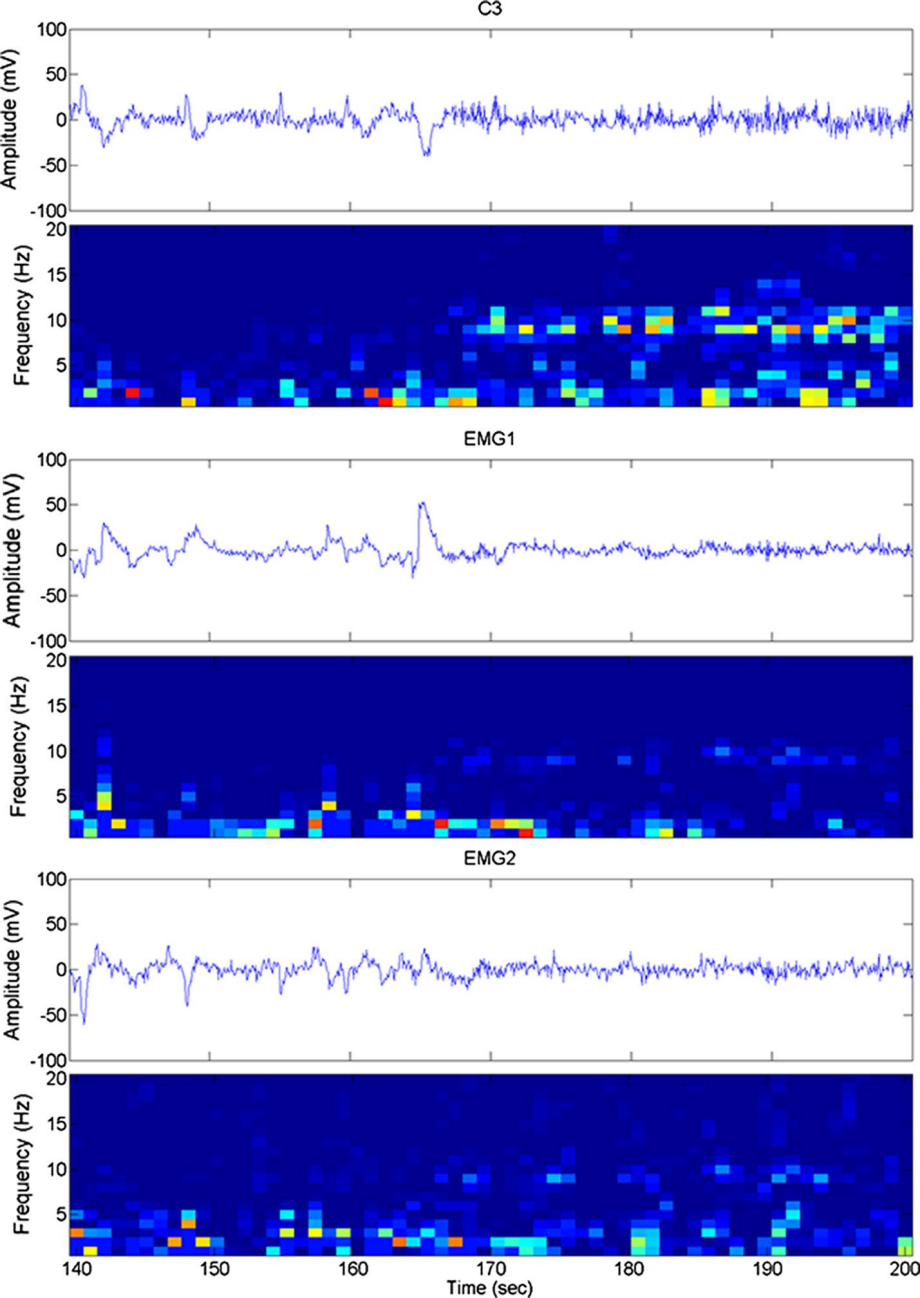
Example of artifact of EEG of the C3 lead and activities of mentalis muscle (EMG1) and masseter muscle (EMG2) accompanied by their time–frequency spectrograms. The trace at the beginning contains numerous muscular responses

To further assess contribution EMGs on the NFT, activities of mentalis muscle (acting for facial expression) and masseter muscle (acting for chewing or jaw movement) were recorded simultaneously with the C3-lead EEG. Figure 7 illustrates the temporal traces and their time–frequency plots within the training. Although the C3 lead presented EMG-related pattern at the beginning of the trace, no obvious activity was observed in the alpha bandwidth in their time–frequency plots. The r values between the C3 lead and EMG of mentalis muscle (0.167 ± 0.019) or masseter muscle (0.153 ± 0.023) were quite low. The results suggest that the C3 lead has a low interference by muscular activity for a training of the alpha band.

### Evaluation of neurofeedback training

**Fig. 8:**
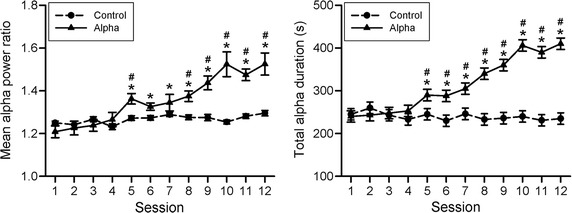
Dynamic changes of the mean alpha power ratio and alpha duration throughout 12 sessions in the two groups. *p < 0.05 when compared with the 1st session; ^#^p < 0.05 when compared with the Control with Bonferroni correction

The MMSE values in all subjects were greater than 25 and had no significant difference before and after the neurofeedback training in the two groups. There was no difference in the baseline activity of the two groups. Figure 8 illustrates the mean alpha power ratio and the total duration of alpha power over the baseline throughout the 12 training sessions. The control group had no obvious change throughout 12 sessions. In the Alpha group, the mean alpha power ratio had no change at the beginning, which may imply a latent trial-and-error learning. Afterwards, it showed a progressive increase throughout the training. The mean alpha power ratio had significant difference in the factors of group (F_1,28_ = 44.552, p < 0.001), session (F_11,308_ = 12.265, p < 0.001), and their interaction (F_11,308_ = 8.352, p < 0.001). There was no significant difference in the control group throughout 12 sessions. In contrast, mean alpha power ratios of the Alpha group showed significant differences at 5th–12th sessions compared to that of its first session, and they also significantly differed from those of the control group at 5th and 8th–12th sessions.

In addition, the Alpha group had no obvious change in total alpha duration at the beginning. The total alpha duration in the Alpha group showed progressive increase throughout the training. The control group had no obvious change throughout 12 sessions. The total duration of alpha rhythm had significant difference in the factors of group (F_1,28_ = 15.486, p < 0.001), session (F_11,308_ = 1.11 * 10^32^, p < 0.001), and the interaction between group and session (F_11,308_ = 1.435 * 10^32^, p < 0.001). Total 8- to 12-Hz durations of 5th–12th sessions in the Alpha group showed significant differences compared to that of its first session, and they also significantly differed from those of the control group.

### Assessment of cognitive function

**Fig. 9:**
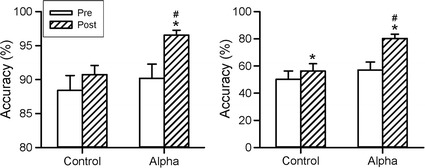
Changes in the accuracies of the backward digit span test (left panel) and word-pair test (right panel) before and after the neurofeedback training of the two groups. *p < 0.05 when compared with the pretest; ^#^p < 0.05 when compared with the Control with Bonferroni correction

Performance of the backward digit span test in the two groups before and after NFT is shown in Fig. 9. Accuracy of the backward digit span test showed a significant difference in the factor of time (F_1,28_ = 14.987, p < 0.001), but not in the factors of group (F_1,28_ = 3.249, p = 0.082) and their interaction (F_1,28_ = 3.313, p = 0.079). The control group had no difference before and after the training. In contrast, the Alpha group had significant increase in accuracy after the training, and the Alpha group after the training had significantly higher accuracy than that of the control group. Furthermore, 13 participants of the Alpha group (86.7%) showed improved accuracy (6.4 ± 1.9; range − 2.2 to 23.2) after NFT, and 11 participants of the control group (73.3%) had improved accuracy (2.3 ± 1.2; range − 5.0 to 10.6) after NFT. Improved accuracy of the Alpha group was significantly higher than the control group (p = 0.042).

The performance of the word-pair test in the two groups before and after NFT is shown in the right panel of Fig. 9. Accuracy of the word-pair test showed significant difference in the factors of group (F_1,28_ = 4.661, p = 0.040), time (F_1,28_ = 54.276, p < 0.001) and their interaction (F_1,28_ = 18.412, p < 0.001). Both groups showed significant increases on accuracy after NFT. Moreover, the Alpha group had significantly higher accuracy than the control group after the training. Furthermore, all participants of the Alpha group (100%) had improved accuracy (23.1 ± 3.7; range 1.25–51.25) after NFT, and 13 participants of the control group (86.7%) showed improved accuracy (6.1 ± 1.5; range − 3.75 to 17.5). Improved accuracy of the Alpha group was significantly higher than the control group (p < 0.001).

## Discussion

A portable system based on low-power wireless Bluetooth technology was developed for NFT of EEG’s alpha rhythm. The proposed portable system utilized a modern smartphone to control the training procedure and visual feedback of 1-channel EEG information. The present study provided evidence that neurofeedback can be implemented in a tiny size of EEG signal device with the mobility of a smartphone. The NFT system increased alpha power and alpha duration throughout the training in the Alpha group exclusively. The Alpha group had significant elevation of memory in terms of the word-pair task and backward digital span task compared to the control group. The findings demonstrate the effect of alpha rhythm on both working memory and episodic memory, which extends the findings on enhancement of working memory and attention in previous studies [9, 10, 26]. These results indicate that memory enhancement is greatly related to activation of alpha rhythm.

Most previous research related to portable or wireless biomedical systems mainly focused on the applications of physiological signal recordings or health monitoring [20, 30]. Compared to these wireless systems, the present study added a feedback module and provided evidence of memory enhancement with a well experimental design. Most previous studies use neurofeedback apparatus with wire connection and considerably bulky size [9, 10, 31]. Our mobile device provides a feasibility with wireless connection and advantage of tiny size for portability. Moreover, both working memory and episodic memory were enhanced in our wireless NFT apparatus, which seems to be comparable to some systems [10, 26] or superior to previous apparatus [9, 11]. According to portability of tiny size and friendly interaction with a mobile device, our NFT system can motivate people to increasingly use in our living environment to enhance cognitive function. With this scenario, participants can receive more training opportunities conveniently in our portable NFT system.

The system showed significant increases in alpha power and duration between the two groups in the 5th–12th sessions (Fig. 8). In our previous study [26], a significant increase in alpha power began in the 8th session. The slight discrepancy between our previous study and the present study may be due to lower threshold to determine alpha rhythm (1.2- vs. 1.5-fold) or short training schedule (12 sessions in 3 or 4 weeks). When we calculated the results using 1.5-fold threshold, there were significant differences in alpha power and duration between the two groups in the 8th–12th session (data not shown). Obviously, a higher threshold delays the occurrence of significant difference between the two groups. The present study advances our understanding of using a lower threshold and intensive training to indicate a better performance in both power and duration of the alpha band. The early remarkable increase in alpha power or duration perhaps creates potential motivation of a user in an NFT.

The wireless neurofeedback system showed a great ability to enhance both alpha rhythm and memory performance. Our device demonstrated its trainability of alpha rhythm within a short period. This is remarkable because there is typically a three- to tenfold higher number of sessions being utilized in clinical therapy [32, 33]. In general, a portable and flexible training apparatus has a potential advantage to be increasingly used in a laboratory environment or in our daily life setting. To motivate the use of NFT in a daily life, our system provides the information of training date and time as well as changes of EEGs for all training sessions. The information may be useful to drive user’s motivation or to trace neural plasticity for clinical studies [23]. As so to ascertain the system on a daily environment, usability of the portable training device in clinical settings needs to be further evaluated.

Most concerns on a portable device will be artifacts in the recording [14, 18, 23]. In the present study, we have illustrated two major kinds of artifacts, i.e., EOG and facial EMGs, during the NFT. Fortunately, the alpha NFT has little influence by these two external interference. These results not only support the recording quality of our portable apparatus but also suggest a successful training on alpha rhythm.

The present study used an 8-bit ADC and showed progressive increases in the duration and power of alpha rhythm in the Alpha group exclusively. The results indicate that specifications of our system are able to provide valuable advantage for alpha neurofeedback training. Similar resolution of an 8-bit ADC has been used in other physiological recordings, such as electrocardiogram [30]. Compared to an 8-bit ADC, higher resolution ADCs, such as 12–24 bits, are available in some applications to extract subtle changes [23]. As more and more 24-bit ADCs appear on the market and propose theoretical sensitivity for the least significant bit of 3 nV, the remaining issue will be to improve the signal-to-noise ratio to be able to take advantage of this decrease in quantization error of the EEG signals. Whether a high-resolution ADC shows a better performance on NFT still remains to be demonstrated.

Previous neurofeedback studies have used within-subject experimental designs with inappropriate control group or absence of the control group [9–11, 15, 16]. They mostly provide the information about individual effects of cognitive performance or memory before and after training or only provide correlations between the alpha power increase and memory enhancement [9, 10]. A sham-controlled group is better to explain the effect of neurofeedback on cognitive function [3, 12, 17]. The current study validate the effect of alpha neurofeedback training on memory in terms of a mixed-subject experimental design with a sham-controlled group. The control group showed no change throughout 12 sessions training. Only the Alpha group revealed progressive and significant increase in alpha power and alpha duration throughout the training. The brain training has a good control here. On the other hand, both groups had no different memory performance before NFT, which implies a good behavioral control in the current study. The Alpha group showed significant memory enhancement compared to the control group after the training. These results suggest absolute enhancement on working and episodic memories in response to alpha NFT.

In the backward digital span task, there was no accuracy change before and after NFT in the control group. However, the control group had significantly increased accuracy in the word pair task after NFT. The phenomenon is similar to our previous study [26]. A possible reason for the phenomenon may be a learning effect in the word pair task. The Alpha group had significant higher accuracy compared to the control group after NFT. Under a sham-controlled design, we can measure true effect of the alpha NFT on memory with a consideration of possible learning or placebo effect.

In terms of alpha power or total alpha duration throughout the 12 training sessions, alpha rhythm had a progressive increase (Fig. 6). The phenomenon supports that the alpha power of a person is trainable. There was no dramatic change in these two indexes at the beginning of the training session, which may a latent trial-and-error learning behavior [26]. The entire training causes significant alpha enhancement, which suggest a brain plasticity taking place during the training. Most interestingly, both working memory by the backward digit span task and episodic memory by the word-pair task had significant improvement in the Alpha group (Fig. 9). Previous studies have indicated that higher alpha rhythm are associated with better memory or cognitive performance [2, 34]. The present study provides more supporting evidence that a portable device for neurofeedback training induces neural plasticity and enhances cognitive functions. A possible mechanism for alpha enhancement on memory may be related to active cortical inhibition before cognitive task [2, 35], which results in increased capacity for learning or storage. Another possibility may be due to substantial alpha rhythm as a selective filter to enhance signal-to-noise ratio of perception and/or attention (i.e., neural efficiency hypothesis) [36], which may lead to a better memory process.

Participants in a neurofeedback paradigm gain significant cognitive improvement or ameliorate clinical symptoms [2, 7, 32, 33]. This portable system enhanced alpha rhythm and elevated memories. The present study provides additional evidence to determine a non-pharmacological alternative intervention on memory enhancement. Brain rhythms, such as sensorimotor rhythm and theta wave, reveal different effects on cognition or clinical syndromes [2, 17, 33]. It will be interesting to extend the system application in various brain rhythms to enhance cognitive functions in healthy subjects or to ameliorate pathological symptoms in patients.

## Conclusion

A portable wireless neurofeedback training system of EEG alpha rhythm was developed and validated in terms of trainability by changes of alpha power and alpha duration and enhancement of working and episodic memory. The portable system may be better to gain more training opportunities in a daily setting to continuously elevate or maintain memory. The device provides an alternative non-pharmacological intervention for memory enhancement.

## Abbreviations

ADC: analog-to-digital converter
ANOVA: analysis of variance
BLE: Bluetooth low energy
EEG: electroencephalogram
EMG: electromyogram
EOG: electrooculogram
FFT: fast Fourier transform
MCU: microcontroller unit
MMSE: Mini-Mental State Examination
NFT: neurofeedback training
